# Integrating Pt nanoparticles with 3D Cu_2-_ *_x_* Se/GO nanostructure to achieve nir-enhanced peroxidizing Nano-enzymes for dynamic monitoring the level of H_2_O_2_ during the inflammation

**DOI:** 10.3389/fimmu.2024.1392259

**Published:** 2024-07-17

**Authors:** Man Shen, Xianling Dai, Dongni Ning, Hanqing Xu, Yang Zhou, Gangan Chen, Zhangyin Ren, Ming Chen, Mingxuan Gao, Jing Bao

**Affiliations:** ^1^ Department of Clinical Laboratory Medicine, Southwest Hospital, Third Military Medical University (Army Medical University), Chongqing, China; ^2^ College of Pharmacy and Laboratory Medicine, Third Military Medical University (Army Medical University), Chongqing, China; ^3^ State Key Laboratory of Trauma, Burn and Combined Injury, Army Medical University, Chongqing, China

**Keywords:** wound inflammation, H2O2 detection, peroxidase (POD) mimetic, electrochemical sensing, Cu2-xSe/GO@Pt

## Abstract

The treatment of wound inflammation is intricately linked to the concentration of reactive oxygen species (ROS) in the wound microenvironment. Among these ROS, H_2_O_2_ serves as a critical signaling molecule and second messenger, necessitating the urgent need for its rapid real-time quantitative detection, as well as effective clearance, in the pursuit of effective wound inflammation treatment. Here, we exploited a sophisticated 3D Cu_2-_ *_x_* Se/GO nanostructure-based nanonzymatic H_2_O_2_ electrochemical sensor, which is further decorated with evenly distributed Pt nanoparticles (Pt NPs) through electrodeposition. The obtained Cu_2-_ *_x_* Se/GO@Pt/SPCE sensing electrode possesses a remarkable increase in specific surface derived from the three-dimensional surface constructed by GO nanosheets. Moreover, the localized surface plasma effect of the Cu_2-_ *_x_* Se nanospheres enhances the separation of photogenerated electron-hole pairs between the interface of the Cu_2-_ *_x_* Se NPs and the Pt NPs. This innovation enables near-infrared light-enhanced catalysis, significantly reducing the detection limit of the Cu_2-_ *_x_* Se/GO@Pt/SPCE sensing electrode for H_2_O_2_ (from 1.45 μM to 0.53μM) under NIR light. Furthermore, this biosensor electrode enables *in-situ* real-time monitoring of H_2_O_2_ released by cells. The NIR-enhanced Cu_2-_ *_x_* Se/GO@Pt/SPCE sensing electrode provide a simple-yet-effective method to achieve a detection of ROS (H_2_O_2_、-OH) with high sensitivity and efficiency. This innovation promises to revolutionize the field of wound inflammation treatment by providing clinicians with a powerful tool for accurate and rapid assessment of ROS levels, ultimately leading to improved patient outcomes.

## Introduction

Reactive oxygen species (ROS) play a vital role in wound inflammation treatment (1). Their reactive and destructive properties enable neutrophils and macrophages to phagocytose, thus aiding in inflammation management (2–4). Hydrogen peroxide (H_2_O_2_) is a major secondary messenger in the treatment of inflammation and a typical ROS molecule in biomedical diagnostics (5, 6). Over the past few years, the preponderance of evidence has established that H_2_O_2_ has a particularly significant impact on the stress/inflammatory response and subsequent tissue/neuronal repair processes (7–9). The inherent characteristics of H_2_O_2_ such as its ease of degradation, long half-life, universal present in all cells, and membrane/tissue permeability - dictate its functional role. Within a certain concentration range, H_2_O_2_ performs multiple functions: (1) it stimulates proliferation of human fibroblasts and vascular endothelial cells; (2) it boosts macrophage inflammatory protein (MIP)-1α production; and (3) it promotes angiogenesis via vascular endothelial growth factor (VEGF) signaling and catalytic effects on keratin-forming cells (2, 10). However, at elevated concentrations, H_2_O_2_ can lead to cell necrosis by triggering pro-apoptotic proteins and ultimately causing cell death. It is evident that the physiological effects of H_2_O_2_ are usually concentration-dependent; hence, there is a pressing need to explore effective methods for accurately detecting H_2_O_2_ concentration in living cells (11).

Sensing technologies such as fluorescence (12), chemiluminescence (13), colorimetry (14), photoelectrochemical sensing (15) and electrochemical sensing (16) have been developed for the detection of H_2_O_2_. Recently, electrochemical sensing technology, which offers the advantages of simple operation, fast response, high sensitivity, and high selectivity has been receiving immense research attention for real-time H_2_O_2_ detection. To address the limitations of expensive and environmentally unstable catalase to the widespread application of sensors, there has been a growing interest in the study of biomimetic electrochemical H_2_O_2_ sensors that possess peroxidase-like activity without the need for enzymes (17, 18).

The advancement of efficient nano-enzymatic materials is paramount for expeditious *in situ* detection of H_2_O_2_ (19). These materials, which range from noble metal nanoparticles like Au (20), Pt (21), Ag (22), etc., to metal oxides or compounds nanostructures such as CeO_2_ (23), TiO_2_ (24), MnO_2_ (25), etc., exhibit exceptional hydrogen peroxidase activity. This activity not only effectively mitigates excess H_2_O_2_ in the physiological environments, but also signifies their vast potential in applications like wound healing and inflammation therapy. Specifically, Pt NPs demonstrate promising applications in H_2_O_2_ electrochemical sensors due to their excellent peroxidase-like activity (26, 27). However, ensuring the uniform anchoring of these metal nanoparticles on smooth electrodes remains a significant challenge for electrochemical detection of H_2_O_2_. Fortunately, numerous studies have established that carbon-based materials can be used as support structures for growing and anchoring metal particles, thereby preventing their aggregation (28). Carbon nanosubstrates such as carbon nanotubes (29), graphene (30) and carbon fibers (31) can even facilitate the spontaneous generation of metal nanoparticles on the substrate, eliminating the need for additional reducing agents. As an example, Tong et al (32). reported a self-terminating chemical deposition method to prepare surfactant-free monodisperse Pt nanoparticle (NP)-modified carbon fiber microelectrodes (Pt-NP/CFE) for electrochemical detection of hydrogen peroxide (H_2_O_2_) released by living cells. This method represents a significant breakthrough in the field of nano-enzymatic materials development, paving the way for more effective and precise *in situ* detection of H_2_O_2_. However, the enhancement of stability and catalytic activity of metal nanoparticles remains a formidable challenge for their implementation in the expeditious and precise detection of trace amounts of H_2_O_2_. To achieve rapid detection of minute amounts of H_2_O_2_, extensive research has been conducted on the development of composite nanoenzymes. Innovative researchers have devised various copper-based nanomaterials, including CuO (33), Cu-MOF (34), and CuS (35), and evaluated their efficacy in enzyme-like catalysis. Copper-based nanomaterials hold high research value for their application in H_2_O_2_ detection. Furthermore, copper-based nanomaterials exhibit unique optical properties. In particular, copper selenide nanocrystals (Cu_2-_ *_x_* Se), as a p-type semiconductor nanomaterial, generate a profusion of photogenerated electron-hole pairs under irradiation of near-infrared light by localized surface plasmon resonance (LSPR) in response to oscillating electromagnetic field interactions of light (36). Moreover, the semiconductor-metal heterojunction formed by the union of copper selenide and metal nanoparticles abbreviates the band gap of the material, thereby augmenting its catalytic activity.

**Scheme 1:**
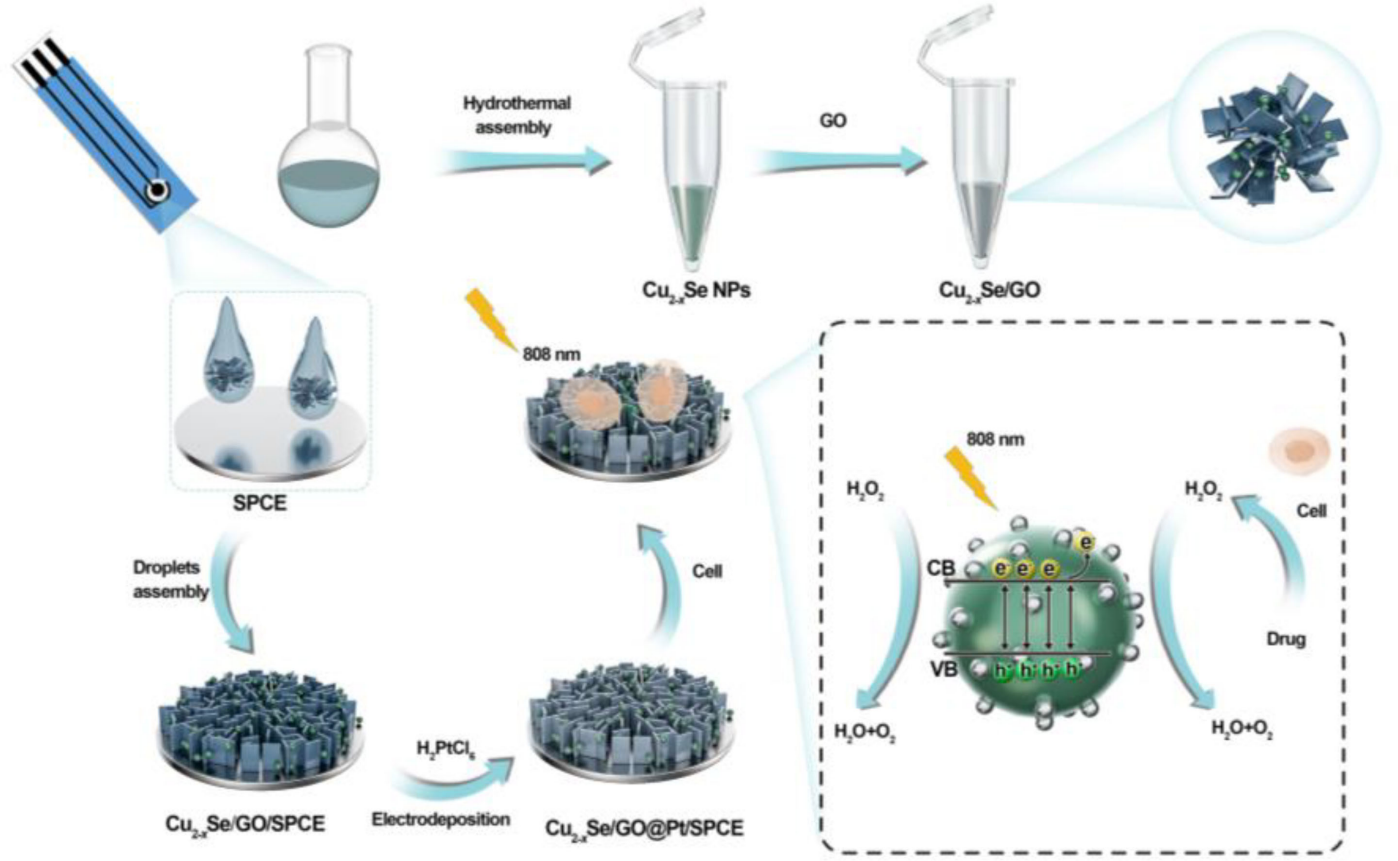
Schematic illustration of the formation of Cu2-xSe/GO@Pt/SPCE electrochemical sensors and its principle for H_2_O_2_ detection.

Herein, we have developed sensing electrodes, made from Cu_2-_ *_x_* Se/GO@Pt composites, for rapid and precise *in situ* detection of H_2_O_2_ (**Scheme 1**). Initially, we employed our previously established method (37) to generate Cu_2-_ *_x_* Se nanospheres containing copper defects. Subsequently, we integrated GO nanosheets as a substrate, ensuring the nanospheres were firmly anchored to the electrodes and facilitating the electrodeposition of Pt nanoparticles. Meanwhile, the three-dimensional structure of the GO nanosheets on the electrode surface not only provides a larger specific surface area, but also offers more efficient electron and ion transport paths, ultimately enhancing the catalytic performance of the sensing electrode. In addition, the unique photoelectric effect of Cu_2-_ *_x_* Se nanorods also plays a crucial role in the enhancement of the sensing electrode’s ability to detect H_2_O_2_. Therefore, the prepared Cu_2-_ *_x_* Se/GO@Pt sensing electrode demonstrates superior electrocatalytic activity towards H_2_O_2_, making it an ideal candidate real-time detection of H_2_O_2_ released from living cells.

## Experimental section

### Reagents and solutions

Polystyrene sulfonic acid (PSS, MW 70 kda), pelenium dioxide (SeO_2_) and Copper sulfate pentahydrate (CuSO_4_·5H_2_O) were all purchased from Aladdin Reagent Co., Ltd. (Shanghai,China). Ascorbic acid (AA), chloroplatinic acid hexahydrate (H_2_PtCl_6_·6H_2_O), glycine (Gly), citric acid (CA), urea, and glutathione (GSH) were obtained from Sigma-Aldrich (St. Louis, USA). Potassium chloride (KCl), potassium ferricyanide (K_3_[Fe(CN)_6_]), potassium hexacyanoferrate(II) (K_3_[Fe(CN)_6_]) and H_2_O_2_ were obtained from Chuandong Chemical Co., Ltd. (Chongqing, China). Phosphate buffered saline (PBS) was purchased from BioInd (Gibco). All chemicals and reagents were used as received without any further purification. All solutions were prepared using ultrapure water (18.2 MΩ). Graphene oxide (GO) powder and Mxene powder were both obtained from Sigma-Aldrich (St. Louis, USA).

### Synthesis of Cu_2-x_Se NPs

Cu_2-_ *_x_* Se NPs were prepared by the previously reported situ reduction method. Briefly, 1.6 mL PSS (10 mg mL^-1^) and 5.5 mL DI water were added into a round-bottom flask which stirred in a 30°C water bath, then 0.3 mL Vc (0.4 M) and 0.1 mL SeO_2_ (0.2 M) were added in sequence, stirring at 30°C for 20 min. A mixed solution of 0.1 mL CuSO_4_·5H_2_O (0.4 M) and 0.4 mL Vc (0.4 M) were added in the next step. Then, the mixed solution was strongly stirred at 45°C for 8h to obtain a dark green dispersion, centrifuged at 12000 rpm for 5 min, and suspended in DI water to obtain Cu_2-_ *_x_* Se solution. Next, the Cu_2-_ *_x_* Se solution freeze dried in vacuum environment, and it was redispersed in DI water (1 mg mL^-1^) at 4°C for later use.

### Fabrication of the Cu_2-x_Se/GO@Pt/SPCE Sensing Electrodes

Firstly, several screen-printed carbon electrodes (SPCE) were prepared, washed with DI water and then dried for use. In a general way, 10 μL Cu_2-_ *_x_* Se NPs (1 mg mL^-1^), 2.5 μL GO (1 mg mL^-1^), and 7.5 μL DI water were uniformly mixed together, and then 10 μL of the mixture (Cu_2-_ *_x_* Se/GO) was dropped on a bare SPCE, followed by drying at 37°C. After that, Pt NPs were electrodeposited on Cu_2-_ *_x_* Se/GO/SPCE in 5 mL mixture of 1 mM H_2_PtCl_6_ solution to obtain Cu_2-_ *_x_* Se/GO@Pt/SPCE. For comparison, the same method was used to prepare Cu_2-_ *_x_* Se/Mxene@Pt/SPCE, Cu_2-_ *_x_* Se @Pt/SPCE, and prepared Cu_2-_ *_x_* Se/SPCE without electrodeposition.

### Characterization of Cu_2-x_Se/GO@Pt/SPCE sensing electrodes

Scanning electron microscopy (SEM) was used to analyze the surface morphology of Cu_2-_ *_x_* Se/GO@Pt/SPCE and the surface distribution of Pt nanoparticles. X-ray photoelectron spectroscopy (XPS) was used to analyze the chemical states of elements on the surface of the prepared sensor electrodes.

### Electrochemical measurements

All the electrochemical tests were performed using the CHI760E electrochemical workstation. The electrodeposition process is carried out for 200 s at a potential of −0.2 V using chronocurrent method (i-t). In order to characterize the electrochemical behavior of Cu_2-_ *_x_* Se/GO@Pt/SPCE, CV and EIS were recorded in a 5 mM [Fe (CN)_6_]^3−/4−^ solution containing 0.1 M KCl. When the electrocatalytic performance of H_2_O_2_ was tested by Cu_2-_ *_x_* Se/GO@Pt/SPCE, CV and i-t were monitored at 0.1 M PBS (pH = 7.4). The test parameters of CV: the operating voltage range is -0.8~ 0.8 V, and the scanning rate is 20-300 mV s^-1^. The linear response of the detection electrode to H_2_O_2_ in the i-t test was performed at an optimal operating voltage of -0.4 V.

### Electrochemical detection of H_2_O_2_ released from cells

The L929 cells (Shanghai Yihe Biotechnology Co., Ltd) were cultured in 5% CO_2_ in 75 cm^2^ culture flask containing MEM medium, 1% penicillin and streptomycin and 10% (v/v) horse serum at 37 °C. Unlike L929 cells, RAW264.7 (Shanghai Yihe Biotechnology Co., Ltd) cells need to be cultured in 75 cm^2^ culture flasks containing DMEM medium, 10% FBS and 1% double antibody at 37 °C and 5% CO2Wash 2 times in PBS (0.01 M, pH=7.4), digest the cells, and centrifuge at 1000 rpm for 3min. Resuspend the cells and counted the cells by calculating instrument. When the current keeps stable, gently stir the cell suspension. Then, the N-Formyl-Met-Leu-Phe (fMLP, Sigma) was added to the cell suspension with different concentration, which can motivate cells generation of H_2_O_2_ and have no interference to the detection of H_2_O_2_. The amperometric current response of H_2_O_2_ in about 7*10^7^ L929 cells or 8*10^7^ RAW 264.7 cells in 10 mL of PBS with the bare SPCE and Cu_2-_ *_x_* Se/GO@Pt/SPCE, and the electrode was recorded at -0.4 V.

## Results and discussion

### Characterization of the As-prepared Cu_2-x_Se/GO@Pt/SPCE sensing electrode

**Figure 1:**
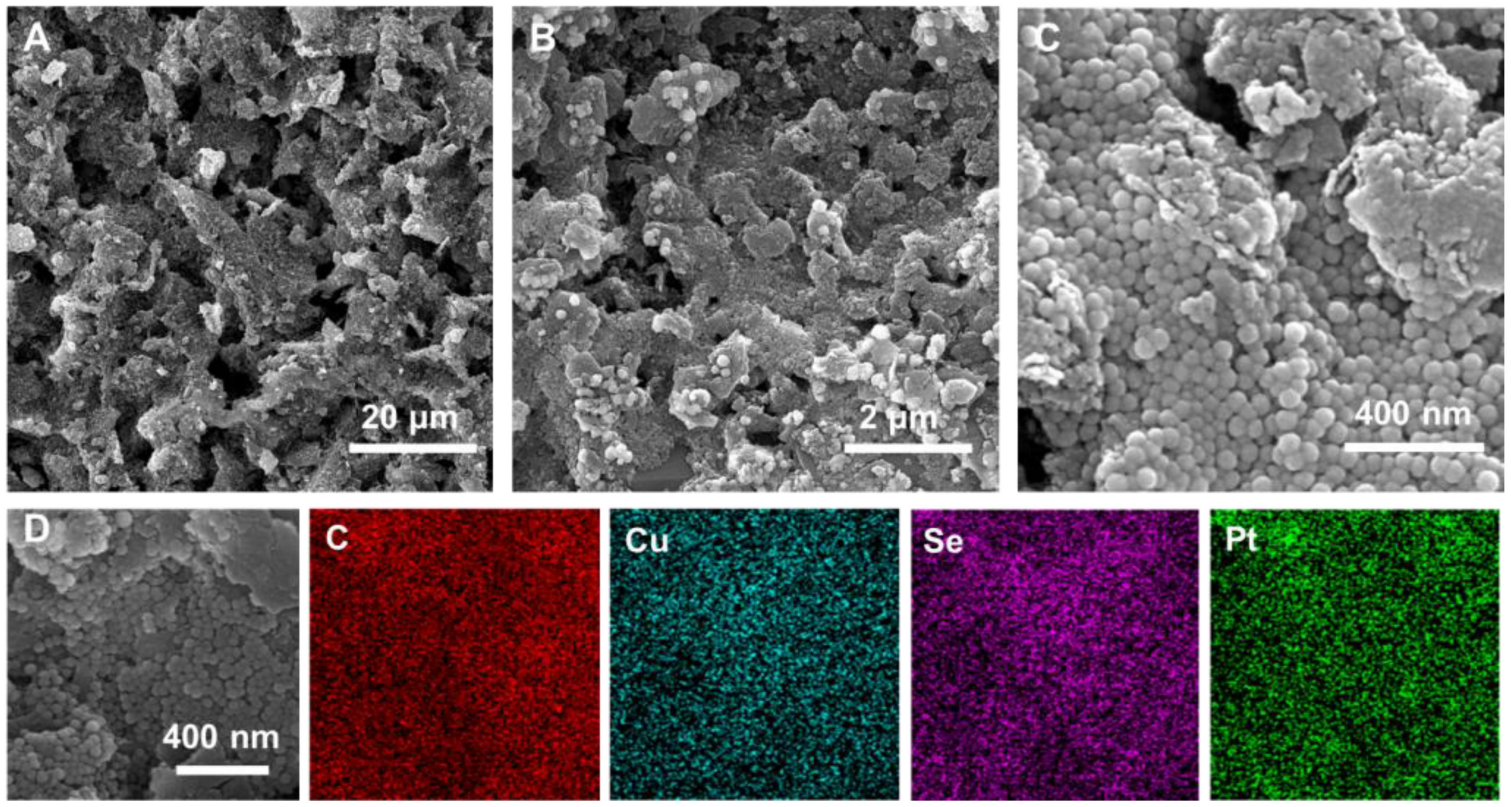
**(A–C)** SEM images of Cu_2-_ *_x_* Se/GO@Pt/SPCE; **(D)** the corresponding elemental mapping images of Cu_2-_ *_x_* Se/GO@Pt/SPCE.

The morphology and structure of the materials and electrodes were characterized by transmission electron microscopy (TEM) and scanning electron microscopy (SEM). As shown in **Supplementary Figure S1**, TEM images of Cu_2-_ *_x_* Se nanospheres proved that they had a uniform spherical morphology with a diameter of about 40 nm and a smooth surface. The SEM images of the bare screen-printed carbon electrodes (SPCE) electrode exhibited that the electrode possesses a rough and porous micro-morphology, which is favorable for modifying the nano-electrode materials (**Supplementary Figures S2A, B**). However, as shown in **Supplementary Figure S2C**, when only Cu_2-_ *_x_* Se NPs were modified on the SPCE, the electrode surface was not completely covered by the nanospheres. In contrast, when the Cu_2-_ *_x_* Se/GO mixture was added dropwise to the electrode, the Cu_2-_ *_x_* Se NPs were evenly distributed across the graphene oxide nanosheets, and the complex completely covered the SPCE surface (**Supplementary Figure S2E**). The incorporation of GO nanosheets provided a reliable three-dimensional (3D) framework for Cu_2-_ *_x_* Se NPs and contributed to their anchoring to the electrodes. SEM images of Cu_2-_ *_x_* Se/GO@Pt/SPCE obtained after modifying Pt nanoparticles (Pt NPs) on Cu_2-_ *_x_* Se/GO/SPCE by electrodeposition were depicted in **Figures 1A–C**. The image results showed that the electrode modified with Pt NPs still presented a 3D porous shape, indicating that the modification of Pt NPs didn’t affect the morphology and structure of GO and Cu_2-_ *_x_* Se NPs. The 3D porous structure formed by GO nanosheets and Cu_2-_ *_x_* Se NPs as a substrate for the electrodeposition of Pt NPs reduced the aggregation of nanoparticles, promoted a robust anchoring of nanoparticles, and provided a reducing agent for the reduction of monomeric Pt. Furthermore, the EDS elemental mapping was carried out to observe the elemental compositions of Cu_2-_ *_x_* Se/GO@Pt/SPCE electrode, in which the Pt signal confirmed the successful loading of the Pt NPs in the Cu_2-_ *_x_* Se/GO structure (**Figure 1D**).

**Figure 2:**
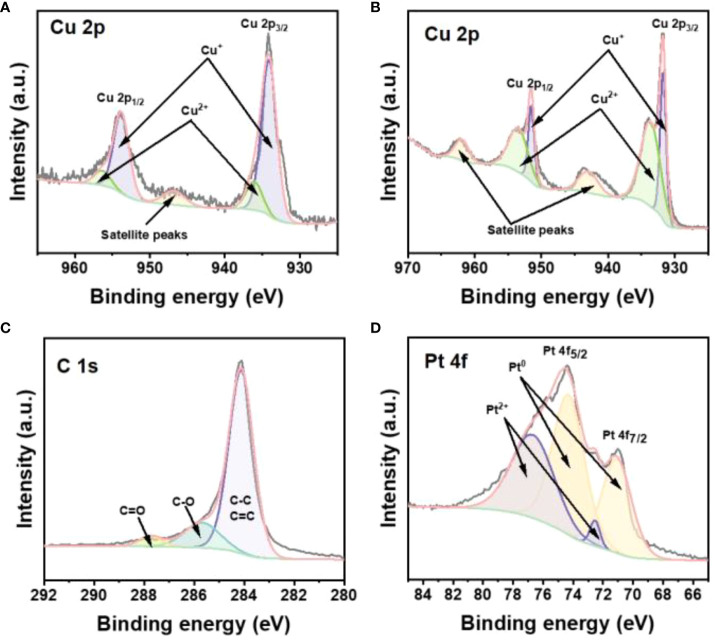
**(A)** The high-resolution XPS spectra for Cu 2p of Cu_2-_ *_x_* Se NPs. The high-resolution XPS spectra for **(B)** Cu 2p, **(C)** C 1s, **(D)** Pt 4f of Cu_2-_ *_x_* Se/GO@Pt/SPCE.

In order to study the elemental chemical composition and valence states of Cu_2-_ *_x_* Se/GO@Pt/SPCE, we characterized the electrode using by X-ray photoelectron spectroscopy (XPS). The XPS spectra revealed the presence of C, Cu, Se, and Pt elements in the Cu_2-_ *_x_* Se/GO@Pt/SPCE (**Supplementary Figure S3A**). The spectra of the Cu 2p orbital indicated the coexistence of both Cu^2+^ and Cu^+^ in Cu_2-_ *_x_* Se NPs (**Figure 2A**) and Cu_2-_ *_x_* Se/GO@Pt/SPCE (**Figure 2B**). Meanwhile, the proportion of Cu^2+^ increased from 23.88% to 31.73% after the deposition of Pt NPs, consistent with the Cu^+^ oxidation process. Compared to Cu_2-_ *_x_* Se NPs, there were obvious negative shifts for the Cu^2+^ and Cu^+^ binding energies of Cu_2-_ *_x_* Se/GO@Pt/SPCE, indicating a strong interaction between Pt NPs and Cu_2-_ *_x_* Se NPs. The high-resolution XPS spectrum of Cu_2-_ *_x_* Se/GO@Pt/SPCE showed peaks of C1s at 284.05, 284.50, and 285.0 eV corresponding to C=C/C-C, C-O, and C=O bonds (**Figure 2C**) (38). Moreover, the Se 3d XPS spectrum of the Cu_2-_ *_x_* Se NPs and Cu_2-_ *_x_* Se/GO@Pt/SPCE exhibited two main peaks (Se 3d_5/2_ and Se 3d_3/2_), indicating that that the valence state of Se element was not affected by the electrodeposition process of Pt NPs (**Supplementary Figures S3B, C**). Additionally, Pt^0^ peaks were observed at the binding energies of 74.4 eV and 71.01 eV, with a Pt^0^ ratio of 72.95% (**Figure 2D**), confirming the successful deposition of Pt NPs. Based on the above analysis, we have successfully prepared a Cu_2-_ *_x_* Se/GO@Pt/SPCE sensing electrode with a unique 3D porous structure, providing a faster conductive network for the electrochemical detection of H_2_O_2_. This advancement paves the way for future applications in sensing technologies.

### Electrochemical characterization of Cu_2-x_Se/GO@Pt/SPCE sensing electrode

**Figure 3:**
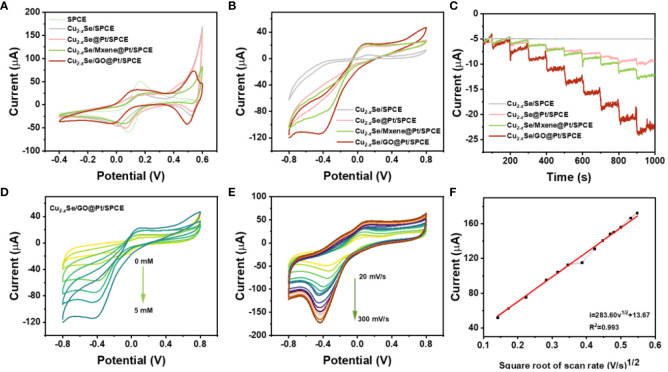
**(A)** CVs of bare SPCE, Cu_2-_ *_x_* Se/SPCE, Cu_2-_ *_x_* Se@Pt/SPCE, Cu_2-_ *_x_* Se/Mxene@Pt/SPCE and Cu_2-_ *_x_* Se/GO@Pt/SPCE in 5 mM [Fe (CN)_6_]^3-/4-^ containing 0.1 M KCl solution at scan rate of 50 mV s^-1^; **(B)** CV curves of Cu_2-_ *_x_* Se/SPCE, Cu_2-_ *_x_* Se@Pt/SPCE, Cu_2-_ *_x_* Se/Mxene@Pt/SPCE and Cu_2-_ *_x_* Se/GO@Pt/SPCE with 5 mM H_2_O_2_ in 0.01 M PBS at 50 mV s^-1^; **(C)** and the i-t curves of the successive additions of H_2_O_2_ with 9 times; **(D)** CV curves of Cu_2-_ *_x_* Se/GO@Pt/SPCE electrode in 0.01 M PBS in the presence of 0~5 mM H_2_O_2_; **(E)** CV curves of Cu_2-_ *_x_* Se/GO@Pt/SPCE with 5 mM H_2_O_2_ in 0.01 M PBS at different scan rates. Scan rate in the range of 20~300 mV s^-1^; **(F)** Linear relationship between the peak current and scan rates.

To evaluate the electrochemical performance of SPCE, Cu_2-_ *_x_* Se/SPCE, Cu_2-_ *_x_* Se@Pt/SPCE, Cu_2-_ *_x_* Se/Mxene@Pt/SPCE and Cu_2-_ *_x_* Se/GO@Pt/SPCE, typical cyclic voltammetry (CV) and electrochemical impedance spectroscopy (EIS) has been applied to thoroughly characterize the electrodes (**Figure 3A**; **Supplementary Figure S4**). As shown in **Figure 3A**, a pair of Fe^2+^/^3+^ redox peaks could be observed on the CV curves of all electrodes in the range of 0-0.2 V. However, the redox peaks of all electrodes except the bare SPCE exhibited wider peak potential differences and lower peak currents. Apparently, these electrodes also exhibited a pair of Cu^2+^/Cu^+^ redox peaks in the range of 0.4-0.6 V, which influenced the peak width and peak current of Fe^2+^/^3+^ redox peak. Among them, Cu_2-x_Se/GO@Pt/SPCE exhibited the highest peak current in the range of 0.4-0.6 V, highlighting its excellent electrochemical performance. Additionally, **Supplementary Figure S4** displays the EIS curves of all the aforementioned electrodes. The EIS curves of the Cu_2-_ *_x_* Se/GO@Pt/SPCE in the line part representing Warburg impedance (Rw) exhibited the steepest slope, indicating that electrolyte ions had the smallest diffusion resistance in the pores of the electrode, thereby facilitating electrochemical reactions. These findings highlight the significance of material design and engineering in enhancing electrochemical performance.

Furthermore, the electrocatalysis of H_2_O_2_ by the prepared electrodes mentioned above was investigated in 10 mL 0.01 M PBS (pH 7.4) system using both CV and i-t methods. The CV curves of each electrode in PBS buffer containing 5 mM H_2_O_2_, as displayed in **Figure 3B**, clearly indicate that the Cu_2-_ *_x_* Se/SPCE without electrodeposition of Pt NPs exhibited no distinct characteristic peak of current response. In contrast, the other three electrodes showed well-defined reduction peaks at -0.32 V (Cu_2-_ *_x_* Se@Pt/SPCE), -0.3 V (Cu_2-_ *_x_* Se/Mxene@Pt/SPCE), and -0.4 V (Cu_2-_ *_x_* Se/GO@Pt/SPCE). These findings convincingly demonstrate that Pt NPs possess a profound catalytic effect on H_2_O_2_. Notably, the Cu_2-_ *_x_* Se/GO@Pt/SPCE exhibited the largest reduction peak current. Subsequently, i-t tests were conducted on the corresponding sensing electrodes at the reduction potentials observed in the CV curves. The i-t curves presented in **Figure 3C** align with the CV curves, offering consistent results. With the continuous addition of 0.1 mM H_2_O_2_, the current signal presented a stepped response, and the Cu_2-_ *_x_* Se/GO@Pt/SPCE displayed the highest response current. Obviously, the above results confirmed that the catalytic reduction of H_2_O_2_ by the sensing electrode was mainly attributed to the Pt NPs on the electrodes. When comparing the two substrate materials, Mxene and GO nanosheets, it is evident that the latter is a more suitable support material for Cu_2-_ *_x_* Se NPs and an effective electrodeposition reducer for Pt NPs, thanks to its constructed three-dimensional porous structure. Furthermore, except for Cu_2-_ *_x_* Se/SPCE, the reduction peak current signals of CV curves of other electrodes gradually increased with increasing H_2_O_2_ concentration (0-5 mM), while the reduction peak potential shifted slightly in the negative direction (**Figure 3D**; **Supplementary Figure S5**).

In order to gain a deeper understanding of the catalytic kinetic process of H_2_O_2_ on the electrode, we conducted CV studies to measure the catalytic current signals of 5 mM H_2_O_2_ on Cu_2-_ *_x_* Se/GO@Pt/SPCE at different scanning rates. As displayed in **Figure 3E**, a gradually increasing scan rate ranging from 20 to 300 mV s^-1^ resulted in an increase in the reduction current. Additionally, a strong linear relationship was observed between the peak current and the square root of the scanning rate (see **Figure 3F**), indicating that the catalytic process is highly dependent on the scan rate. The equation describing this relationship is provided below:


$$i(\mu A)\;=\;283.60v^{\frac{1}{2}}(V/s^{1/2})+\;13.67\;\left(R^{2\;=}0.993\right)$$

By scrutinizing the catalytic reduction reaction of H_2_O_2_ on Cu_2-_ *_x_* Se/GO@Pt/SPCE, we’ve ascertained that it’s an irreversible process, controlled by diffusion. This observation underscores the exceptional electrocatalytic activity of Cu_2-_ *_x_* Se/GO@Pt/SPCE. Consequently, we selected this material for a more detailed examination of its sensitivity in detecting H_2_O_2_.

To optimize detection parameters, we conducted chronoamperometry (i-t) to study the effect of Cu_2-_ *_x_* Se/GO@Pt/SPCE on the catalytic performance of H_2_O_2_ at different operating potentials (-0.36 V~-0.4 V). As depicted in **Figure S6A**, as the voltage increased from -0.36 V to -0.4 V, the response current of Cu_2-_ *_x_* Se/GO@Pt/SPCE to H_2_O_2_ rises gradually. However, upon further voltage increment, the response current does not continue to climb, and even slightly decreased. Clearly, this suggests that Cu_2-_ *_x_* Se/GO@Pt/SPCE reaches peak catalytic performance for H_2_O_2_ at -0.4V. Therefore, -0.4V was chosen as the operating potential for the subsequent experiments.

**Figure 4:**
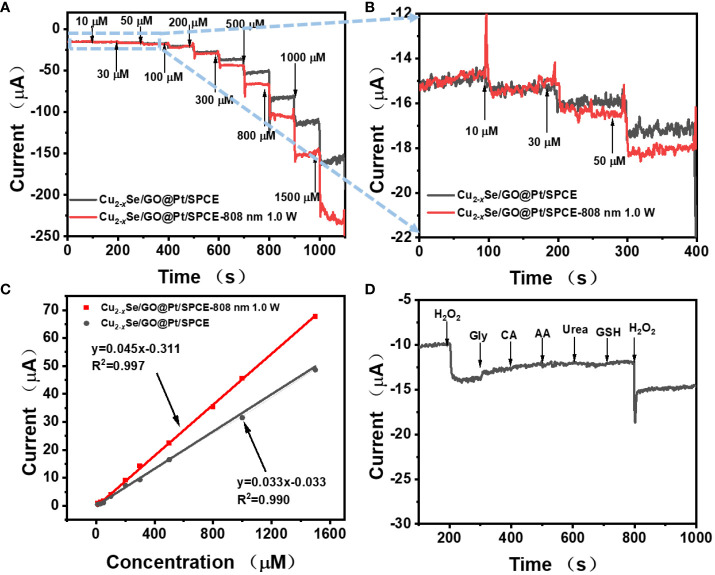
**(A)** Amperometric response of Cu_2-_ *_x_* Se/GO@Pt/SPCE at potential of−0.4 V (without laser and with 808 nm laser) with the successive addition of various amounts of H_2_O_2_; **(B)** the magnified parts of **(A)** at low H_2_O_2_ concentrations; **(C)** Calibration curves between electrocatalytic current of Cu_2-_ *_x_* Se/GO@Pt/SPCE and H_2_O_2_ concentration within a range of 10−1500 μM without laser (gray line) and with 808 nm laser (red line); **(D)** The study of the selectivity, amperometric responses of the Cu_2-_ *_x_* Se/GO@Pt/SPCE to the successive dropwise additions of 0.1 mM H_2_O_2_ and 1 mM interfering species (Gly, CA, AA, Urea and GSH).

Additionally, we evaluated the linear relationship between Cu_2-_ *_x_* Se/GO@Pt/SPCE’s response to H_2_O_2_ at an operating potential of -0.4V through i-t measurements. At the same time, considering the optical characteristics of Cu_2-_ *_x_* Se NPs, the method of near infrared illumination was used to improve the catalytic effect of Cu_2-_ *_x_* Se/GO@Pt/SPCE on H_2_O_2_. In addition, in order to investigate the effect of the content of Pt NPs on the electrochemical performance of the sensor electrode enhanced by NIR, we tested the i-t curves of the sensor electrode with different deposition times. **Supplementary Figure S7A** displays the amperometric response of Cu_2-_ *_x_* Se/GO@Pt/SPCE with successive injection of 100 μM H_2_O_2_ at different deposition times with 808 nm laser. When the deposition time is greater than 200s, the growth of current signal tends to be stable. Thus, 200 s of deposition time was used for subsequent experiments. As **Figure 4A** clearly illustrates, regardless of whether 808 nm laser irradiation was used, the continuous addition of H_2_O_2_ solution with increasing concentration to a 10 mL 0.01M PBS solution led to a rapid step-type response of the reduction current of Cu_2-_ *_x_* Se/GO@Pt/SPCE. This response reached a stable state within a few seconds (<5s), highlighting the electrode’s exceptional response speed and its ability to catalyze H_2_O_2_ rapidly. Obviously, under 808 nm laser irradiation, the Cu_2-_ *_x_* Se/GO@Pt/SPCE displayed a significantly larger response current to H_2_O_2_, indicating that the laser could enhance the electrode’s catalytic effect on H_2_O_2_. Moreover, **Figure 4B** presents the i-t curves of the low concentration region (10~50 μm) in **Figure 4A**, in which the improvement of H_2_O_2_ catalytic performance of Cu_2-_ *_x_* Se/GO@Pt/SPCE by 808 nm laser at low concentration could be more clearly seen. **Figure 4C** further illustrates the linear relationship curves between the current signal and H_2_O_2_ concentration, both without and with 808 nm laser irradiation. The corresponding linear fitting equation is as follows: $i\left(\mu A\right)=0.033C_{H_{2}O_{2}}\left(\text{μM}\right)-0.033\left(R^{2}=0.990\right)$ without laser) and $i\left(\mu A\right)=0.045C_{H_{2}O_{2}}\left(\text{μM}\right)-0.311\left(R^{2}=0.997\right)$ , with 808 nm laser). Based on the above linear fitting equation, the detection limits without laser irradiation and under 808 nm laser irradiation could be calculated as 1.45μM and 0.53 μM, respectively (LOD=3σSd) . Compared to the other electrochemical sensors and other detection methods in **Supplementary Table S1**, a relatively low detection limit is obtained at Cu_2-_ *_x_* Se/GO@Pt/SPCE. findings underscore the critical role of near-infrared illumination in enhancing the H_2_O_2_ catalytic performance of Cu_2-_ *_x_* Se/GO@Pt/SPCE. This exceptional enhancement effect under NIR light is primarily attributed to the local surface plasmon resonance (LSPR) of Cu_2-_ *_x_* Se NPs. which generated more charge carriers on the electrode surface under laser irradiation, and these charge carriers then reacted with H_2_O_2_ to produce a larger response current.

In practical applications, the selectivity and reproducibility of the sensor are paramount considerations. We evaluated these factors by monitoring the interference of several potential interfering substances (glycine (Gly), citric acid (CA), ascorbic acid (AA), urea, glutathione (GSH)) on the detection of H_2_O_2_ using Cu_2-_ *_x_* Se/GO@Pt/SPCE with i-t curve. As illustrated in **Figure 4D**, even at higher concentrations (1 mM), Gly, CA, AA, urea and GSH did not produce any discernible amperometric response when compared to H_2_O_2_ (100 μM). These results suggested the remarkable selectivity for H_2_O_2_ electrochemical detection. Furthermore, we measured the current response of five Cu_2-_ *_x_* Se/GO@Pt/SPCE sensing electrodes to 300 μM H_2_O_2_ with i-t curves and repeated the test six times for each electrode (**Supplementary Figure S8**). The relative standard deviation (RSD) among electrodes was calculated to be 8.27%, indicating excellent reproducibility of the sensor.

### Detection of H_2_O_2_ released from living cells at Cu_2-x_Se/GO@Pt/SPCE

**Figure 5:**
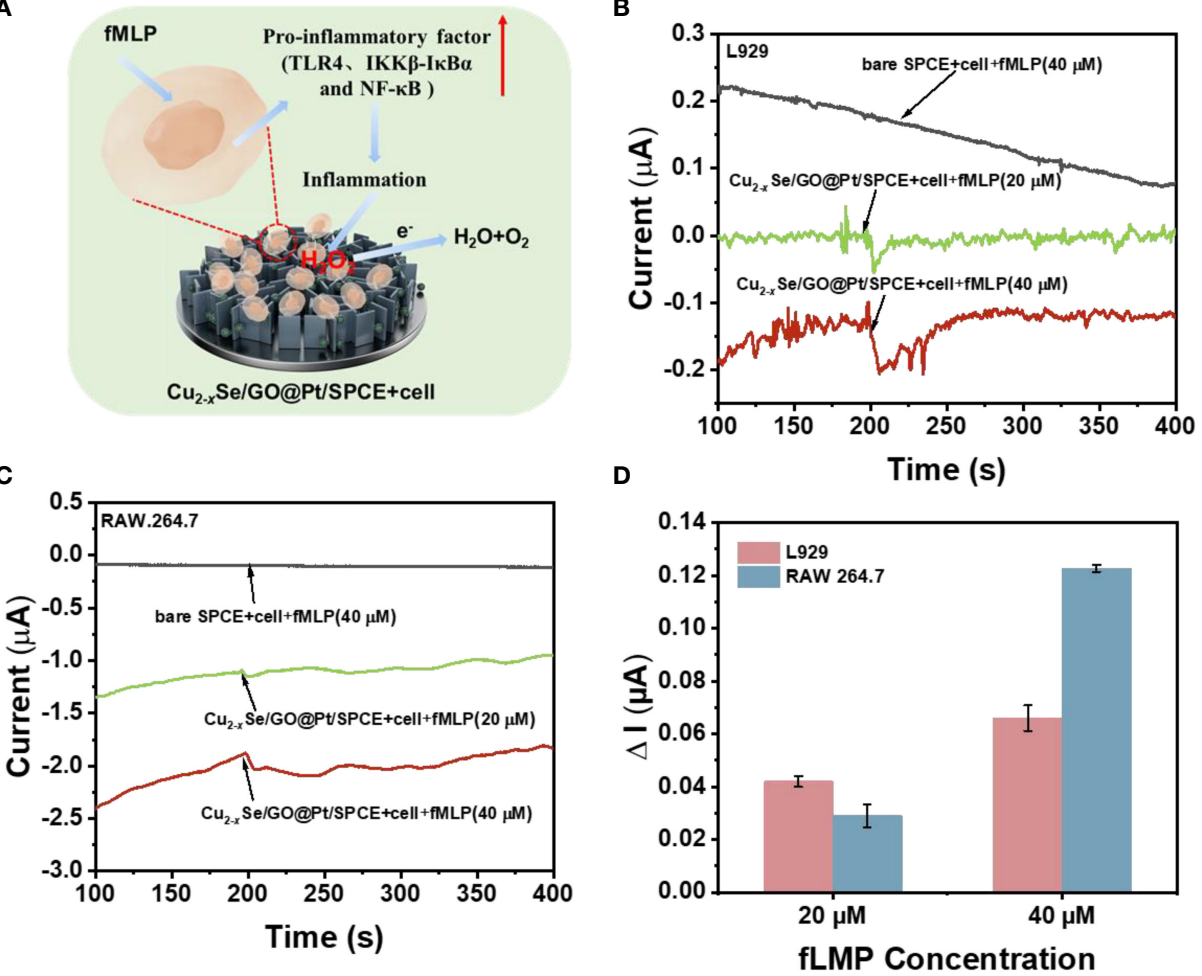
**(A)** Demonstration of a pathway for H_2_O_2_ production by human cells and a scheme for the timely capture of H_2_O_2_ by sensing electrodes in response to drug stimulation; **(B)** i-t response of the Cu_2-_ *_x_* Se/GO@Pt/SPCE sensing platform to the different concentration addition of fMLP stimulation with and without living L929 cells (7*10^7^) at an applied potential of -0.40 V in 0.01 M PBS buffer (pH=7.4); **(C)** i-t response of the Cu_2-_ *_x_* Se/GO@Pt/SPCE sensing platform to the different concentration addition of fMLP stimulation with and without living RAW 264.7 cells (8*10^7^) at an applied potential of -0.40 V in 0.01 M PBS buffer (pH=7.4); **(D)** the corresponding average value of the i-t responses (n=3).

The concentration of H_2_O_2_ in living cells is a significant biological parameter associated with wound inflammation treatment. Therefore, in this study, L929 cells and RAW 264.7 cells were selected as model cells, and the fLMP drug was used to stimulate the release of H_2_O_2_ within them. The microscopic images of L929 cells and RAW 264.7 cells displayed in **Supplementary Figure S9**, L929 cells exhibit fibroblast-like characteristics and grow close to the cell wall, while RAW 264.7 cells possess a small, translucent, and rounded morphology. **Figure 5A** provides a schematic diagram demonstrating how fLMP stimulates cells to produce H_2_O_2_, which is then rapidly detected followed by rapid detection of H_2_O_2_ by Cu_2-_ *_x_* Se/GO@Pt/SPCE. By utilizing Cu_2-_ *_x_* Se/GO@Pt/SPCE as the sensing electrode, the cells were stimulated to release H_2_O_2_ upon the addition of the fLMP drug to two distinct cell suspensions, and the signal of H_2_O_2_ was then determined by the i-t measurement (**Figures 5B, C**). When compared to a bare SPCE electrode, the Cu_2-_ *_x_* Se/GO@Pt/SPCE sensing electrode exhibited significant reduction currents for H_2_O_2_ produced under different concentrations of fLMP (20 μM, 40 μM) stimulation within both cell suspensions, suggesting that the Cu_2-_ *_x_* Se/GO@Pt nano-mimetic enzyme exhibits remarkable catalytic activity towards H_2_O_2_. The reduction currents generated by the bare SPCE electrode and the Cu_2-_ *_x_* Se/GO@Pt/SPCE sensing electrode stimulated with the same concentration of fLMP (40 μM) in both cell suspensions are shown in **Supplementary Figure S10**, and there is a strong significant difference between the two sets of data, which further suggests that the catalytic reduction of H_2_O_2_ is mainly attributed to the Cu_2-_ *_x_* Se/GO@Pt nanomimetic enzyme. In particular, the i-t curve of the Cu_2-_ *_x_* Se/GO@Pt/SPCE retained a significant H_2_O_2_ response peak even when a lower concentration (8 μM) of fLMP solution was added to the L929 cell suspension (**Supplementary Figure S11**). This further highlights the outstanding catalytic activity of the Cu_2-_ *_x_* Se/GO@Pt nanomimetic enzyme. The magnitude of the current response across the curves demonstrated that the amount of H_2_O_2_ produced by the cells increased in direct correlation with the concentration of fLMP. Additionally, as shown in **Figure 5D**, the magnitudes of the reaction currents in both cell suspensions were 0.046 μA, 0.066 μA (L929) and 0.029 μA, 0.123 μA (RAW 264.7) when fLMP was added in the amounts of 20 μM and 40 μM, respectively, indicating that fLMP promotes the dose-dependent release of H_2_O_2_ from the cells. These findings suggest that Cu_2-_ *_x_* Se/GO@Pt offers immense potential as a biomimetic enzyme material for determining H_2_O_2_ released from living cells. This novel approach could significantly enhance our understanding of the mechanisms of ROS action in wound inflammation and pave the way for simplified monitoring of ROS dynamics in inflammatory environments.

## Conclusions

In conclusion, our work has resulted in the successful synthesis of a NIR-enhanced Cu_2-_ *_x_* Se/GO@Pt/SPCE sensing electrode. This non-enzymatic current electrode demonstrates high selectivity, a low detection limit (0.53 μM) and wide linear range (10 μM-1500 μM) for H_2_O_2_ detection. The GO nanosheets not only build a solid three-dimensional structure for the anchoring of Cu_2-_ *_x_* Se nanorods and the electrodeposition of Pt NPs, but also provide additional ion/electron transport channels for electrochemical reactions. The localized surface plasma effect of Cu_2-_ *_x_* Se further enhances the catalytic effect of the electrode on H_2_O_2_ under near-infrared illumination conditions by promoting the separation of photogenerated electron-hole pairs in the Cu_2-_ *_x_* Se@Pt heterojunction. Furthermore, the Cu_2-_ *_x_* Se/GO@Pt/SPCE enables rapid capture of the reduction current signal of H_2_O_2_ release from living cells in L929 and RAW 264.7 cell suspension, allowing for *in situ* real-time detection. Overall, this Cu_2-_ *_x_* Se/GO@Pt/SPCE sensing electrode offers superior performance, cost-effectiveness, simple preparation steps, and high reproducibility, making it a promising candidate for real-time *in situ* detection of biomolecules. This innovation holds the potential to transform the field of wound inflammation treatment by offering clinicians a robust method for promptly and precisely assessing ROS levels, ultimately enhancing patient outcomes.

## Data availability statement

The original contributions presented in the study are included in the article/**Supplementary Material**, further inquiries can be directed to the corresponding author/s.

## Author contributions

MS: Writing – review & editing, Writing – original draft, Methodology, Formal Analysis, Data curation, Conceptualization. XD: Writing – review & editing, Writing – original draft, Methodology, Data curation. DN: Writing – original draft, Methodology. HX: Writing – original draft, Methodology. YZ: Writing – original draft, Investigation. GC: Writing – original draft, Formal Analysis, Data curation. ZR: Writing – original draft, Data curation. MC: Writing – review & editing, Conceptualization. MG: Writing – review & editing, Funding acquisition, Conceptualization. JB: Project administration, Methodology, Writing – review & editing, Writing – original draft, Funding acquisition, Formal Analysis, Conceptualization.
