# Supplementary material for: Integrating Pt nanoparticles with 3D Cu2- x Se/GO nanostructure to achieve nir-enhanced peroxidizing Nano-enzymes for dynamic monitoring the level of H2O2 during the inflammation
Source: Front Immunol. 2024 Jul 17;15:1392259. doi: 10.3389/fimmu.2024.1392259 (PMC11288797; doi:10.3389/fimmu.2024.1392259)
Supplement: Supplementary file 1 [file DataSheet_1.pdf]

Supporting Information

Integrating Pt nanoparticles with 3D $Cu_{2-x}Se$/GO Nanostructure to Achieve NIR-enhanced Peroxidizing Nano-enzymes for Dynamic Monitoring the Level of H_2O_2 during the Inflammation

Man Shen[1†], Xianling Dai[1†], Dongni Ning[2], Hanqing Xu[1], Yang Zhou[1], Gangan Chen[1], Zhangyin Ren[1], Ming Chen[1,2,3]*, Mingxuan Gao[1]*, Jing Bao[1]*

[1]*Department of Clinical Laboratory Medicine, Southwest Hospital, Third Military Medical University (Army Medical University), Chongqing 400038, P. R. China;*

[2]*College of Pharmacy and Laboratory Medicine, Third Military Medical University (Army Medical University), Chongqing 400038, China;*

[3]*State Key Laboratory of Trauma, Burn and Combined Injury, Army Medical University, Chongqing 400038, P. R. China.*

[†]*Man Shen, Xianling Dai and Zhiyu Wu contributed equally to the writing of manuscript.*

***Corresponding author. E-mail addresses: chming1971@126.com (M. Chen), mingxuan_gao@163.com (M. Gao), baojing_1991@163.com (J. Bao).**

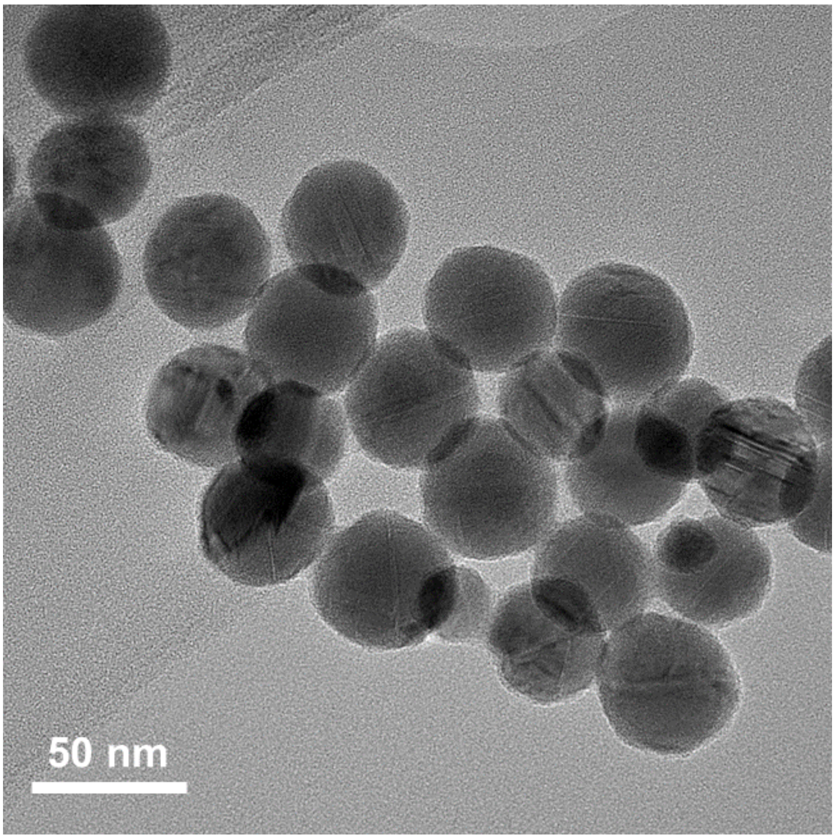

Fig. S1 TEM image of $Cu_{2-x}Se$ NPs.

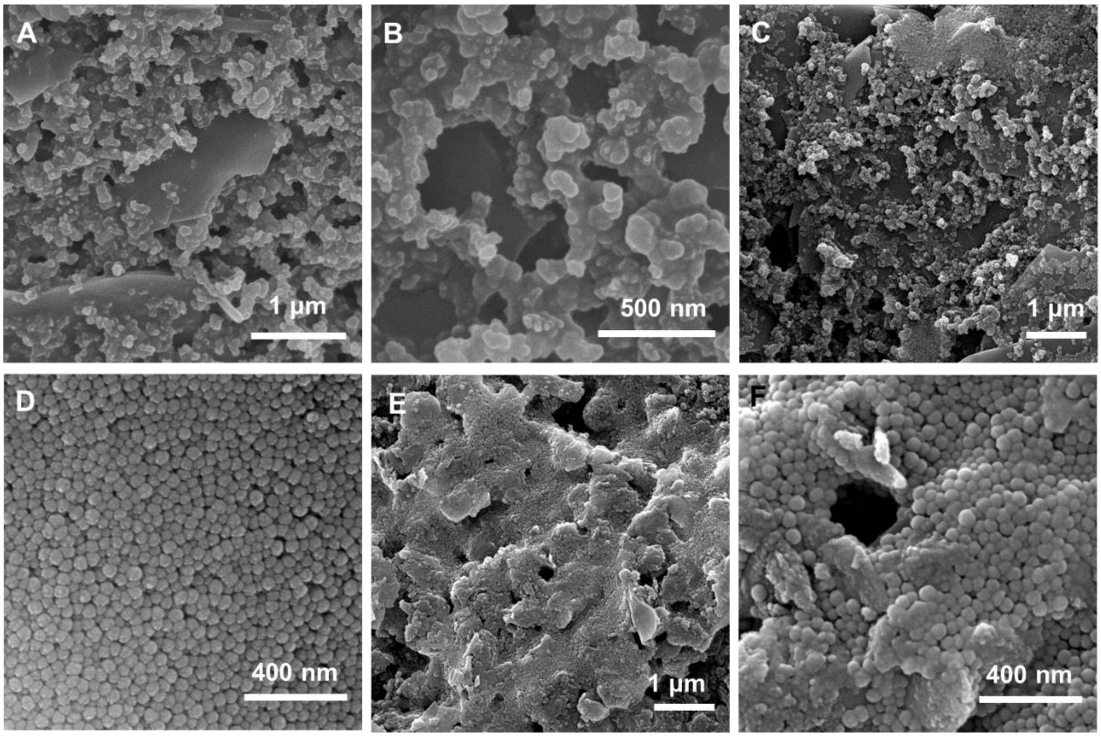

Fig. S2 SEM images of (A)-(B) bare SPCE; (C)-(D) $Cu_{2\text{-}x}Se$/SPCE; (E)-(F) $Cu_{2\text{-}x}Se$/GO/SPCE.

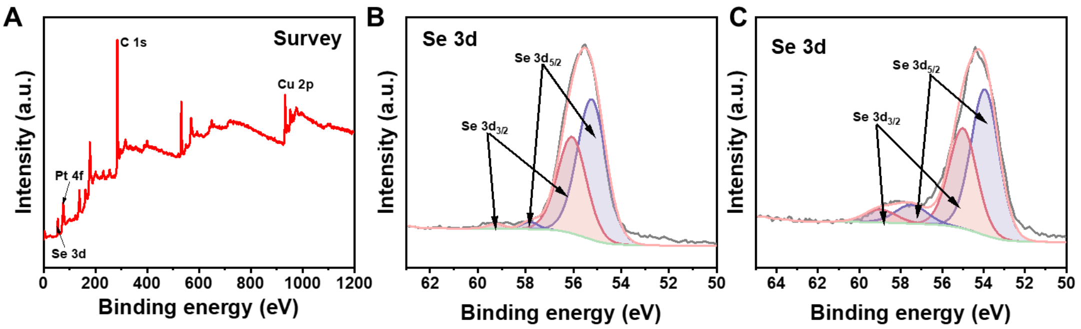

Fig. S3 (A) The XPS survey of $Cu_{2-x}Se$/GO@Pt/SPCE; the high-resolution XPS spectra for Se 3d of (B) $Cu_{2-x}Se$ NPs; (C) $Cu_{2-x}Se$/GO@Pt/SPCE.

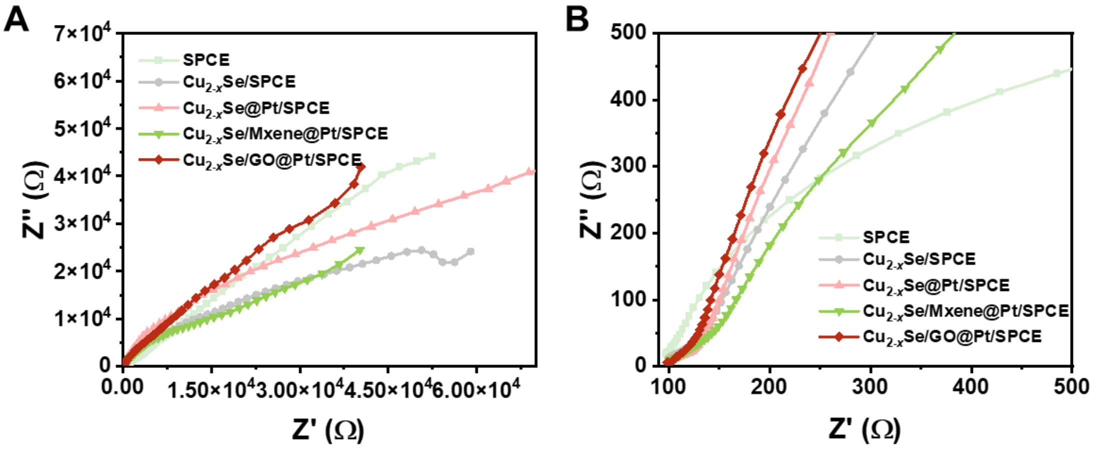

Fig. S4 (A) EISs of bare SPCE, $Cu_{2-x}Se$, $Cu_{2-x}Se$@Pt/SPCE, $Cu_{2-x}Se$/Mxene@Pt/SPCE and $Cu_{2-x}Se$/GO@Pt/SPCE in 5 mM $[Fe\ (CN)_6]^{3-/4-}$ containing 0.1 M KCl solution at scan rate of 50 mV s^{-1}; (B) the magnified parts of (A) at high-frequency zone.

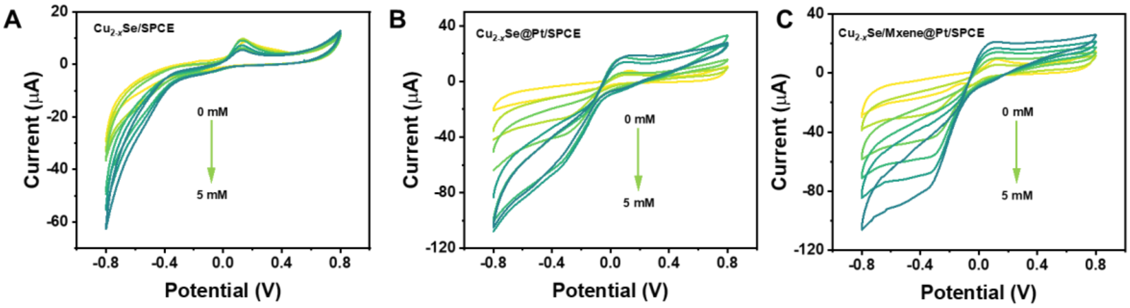

Fig. S5 CV curves of (A) $Cu_{2-x}Se$/SPCE; (B) $Cu_{2-x}Se$@Pt/SPCE; (C) $Cu_{2-x}Se$/Mxene@Pt/SPCE in 0.01 M PBS in the presence of 0~5 mM H_2O_2.

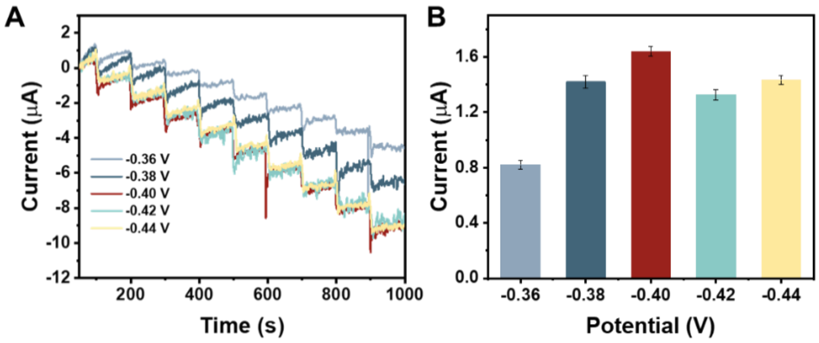

Fig. S6 (A) i-t curves of $Cu_{2-x}Se$/GO@Pt/SPCE with successive injection of 0.1 mM H_2O_2 at various potentials; (B) the corresponding fitting histogram of (A), error bars are the standard error of the mean (n=5).

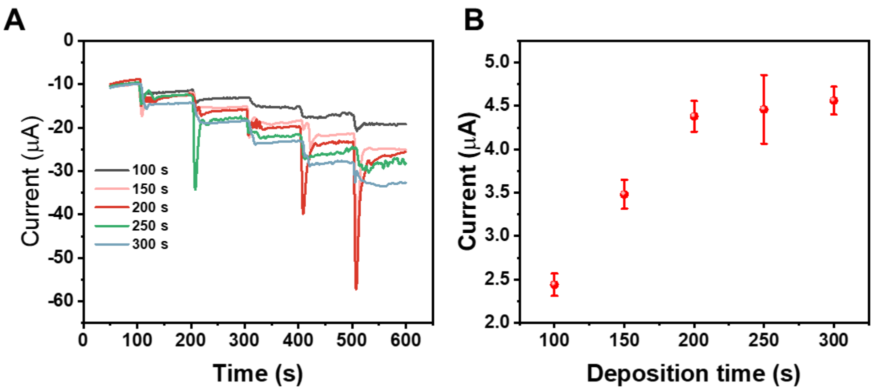

Fig. S7 (A) i-t curves of $Cu_{2-x}Se$/GO@Pt/SPCE with successive injection of 0.1 mM H_2O_2 at different deposition times (100 s, 150 s, 200 s, 250 s and 300 s) with 808 nm laser in 0.01M pH 7.4 PBS solution; (B) the corresponding fitting scatter diagram of (A). Error bars are the standard error of the mean (n=5).

Table S1 Performance comparison of various modified H_2O_2 sensors

Materials or electrodes	Sensing technologies	Linear detection range (μM)	LOD (μM)	Ref.
AuNPs/n-GaN	Electrochemical	40-1000	10	[1]
CuO-CeO_2/MXene/GCE	Electrochemical	5-125	1.67	[2]
Ce-MOF	Colorimetric	4000-16000	10	[3]
MoS_2/CC	Electrochemical	5-3000	1.0	[4]
MnO_2@Ag@PMBA	SERS	10-100	7.44	[5]
Au@Ag nanocubes	Colorimetric	10-1000	0.60	[6]
TMB–Fe_3O_4@AuNPs	Colorimetric	40-5500	11.1	[7]
Fe/$CuSn(OH)_6$	Colorimetric	30-1000	9.49	[8]
$Cu_{2-x}Se$/GO@Pt/SPCE	Electrochemical	10-1500	0.53	This work

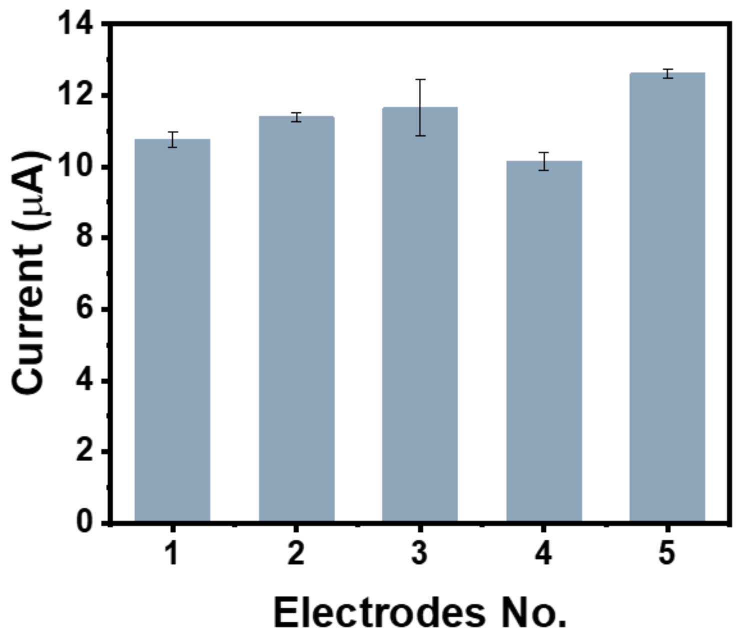

Fig. S8 The reproducibility of $Cu_{2-x}Se$/GO@Pt/SPCE for H_2O_2 detection.

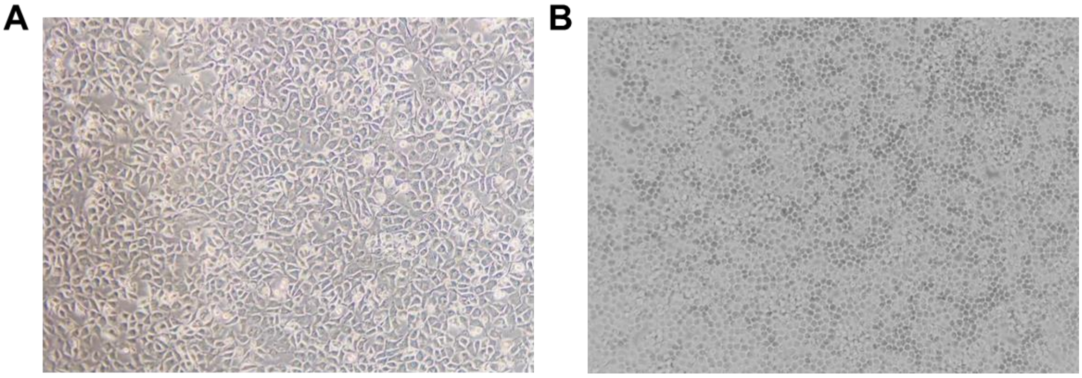

Fig. S9 Micrograph of (A) L929 cells; (B) RAW 264.7 cells.

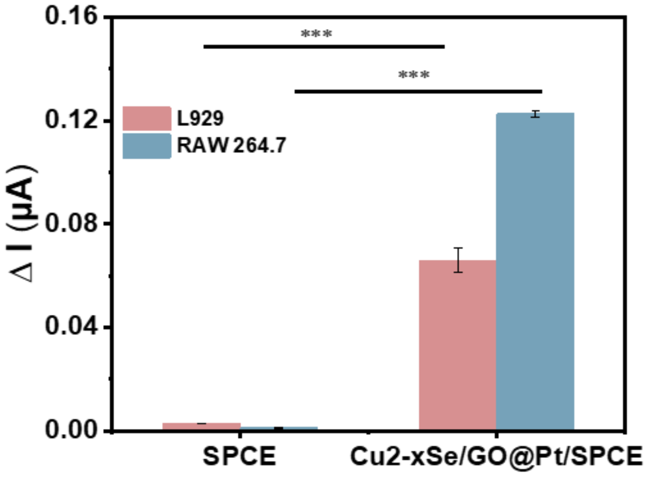

Fig. S10 the average value of the i-t responses (n=3) for bare SPCE and $Cu_{2-x}Se$/GO@Pt/SPCE sensor in different living cell suspensions. Asterisks represent the statistically significant differences (*$p < 0.001$).**

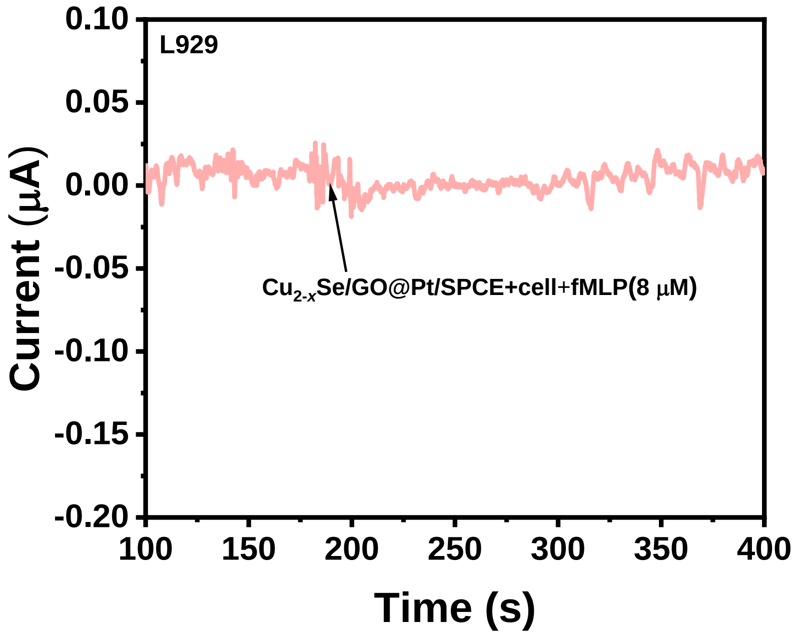

Fig. S11 i-t response of the $Cu_{2\text{-}x}$Se/GO@Pt/SPCE sensing platform to the 8 μM addition of fMLP stimulation with living L929 cells with $7*10^7$ cells at an applied potential of -0.40 V in 0.01 M PBS buffer (pH=7.4)

Reference

1. Ma, C.; Yang, C.; Zhang, M., A Novel Electrochemical Hydrogen Peroxide Sensor Based on AuNPs/n-Type GaN Electrode. *Chemistry Letters* 2020, *49* (6), 656-658.
2. Zhou, K.; Li, Y.; Zhuang, S.; Ren, J.; Tang, F.; Mu, J.; Wang, P., A novel electrochemical sensor based on CuO-CeO2/MXene nanocomposite for quantitative and continuous detection of H2O2. *Journal of Electroanalytical Chemistry* 2022, *921*.
3. Wei, X.; Li, Y.; Qi, S.; Chen, Y.; Yin, M.; Zhang, L.; Tian, X.; Gong, S.; Wang, F.; Zhu, Y.; Liu, Y.; Qiu, J.; Xu, D., Ce-MOF Nanosphere as Colorimetric Sensor with High Oxidase Mimicking Activity for Sensitive Detection of H2O2. *Journal of Inorganic and Organometallic Polymers and Materials* 2022, *32* (9), 3595-3600.
4. Du, H.; Zhang, X.; Liu, Z.; Qu, F., A supersensitive biosensor based on MoS2 nanosheet arrays for the real-time detection of H2O2 secreted from living cells. *Chemical Communications* 2019, *55* (65), 9653-9656.
5. Shen, Y.; Xin, Z.; Hu, X.; Wang, N.; Liu, S.; Wang, J., Dual stimulus-responsive core-satellite SERS nanoprobes for reactive oxygen species sensing during autophagy. *Talanta* 2022, *250*, 123712.
6. Yeh, I. H.; Tadepalli, S.; Liu, K. K., Au@Ag nanostructures for the sensitive detection of hydrogen peroxide. *Sci Rep* 2022, *12* (1), 19661.
7. Liu, Q.; Tang, P.; Xing, X.; Cheng, W.; Liu, S.; Lu, X.; Zhong, L., Colorimetry /SERS dual-sensor of H(2)O(2) constructed via TMB-Fe(3)O(4)@ AuNPs. *Talanta* 2022, *240*, 123118.
8. Liu, H.; Ding, Y.-N.; Yang, B.; Liu, Z.; Zhang, X.; Liu, Q., Iron Doped CuSn(OH)6 Microspheres as a Peroxidase-Mimicking Artificial Enzyme for H2O2 Colorimetric Detection. *ACS Sustainable Chemistry & Engineering* 2018, *6* (11), 14383-14393.
